# Medical students' perceptions of the educational environment at an Iranian Medical Sciences University

**DOI:** 10.1186/1472-6920-10-87

**Published:** 2010-11-29

**Authors:** Teamur Aghamolaei, Ismaeil Fazel

**Affiliations:** 1 Public Health Department, Health School, Hormozgan University of Medical Sciences, Bandar Abbas, Iran; 2 English Department, Medical School, Hormozgan University of Medical Sciences, Bandar Abbas, Iran

## Abstract

**Background:**

Students' perceptions of their educational environment have a significant impact on their behavior and academic progress. The aim of this study was to assess the perceptions of medical students concerning their educational environment at Hormozgan University of Medical Sciences in Iran.

**Methods:**

In this cross-sectional study, questionnaires were distributed to 210 medical students and 182 were analyzed (response rate = 86.6%); twenty-eight questionnaires were excluded because they were incomplete or unreturned for analysis. Data were collected using a DREEM questionnaire which comprised 50 items based on the Likert scale (scores could range from 0 to 200). There were five domains to the questionnaire including students' perceptions of learning, students' perceptions of teachers, students' academic self-perceptions, students' perceptions of atmosphere and students' social self-perceptions. Data were analyzed using SPSS16 software.

**Results:**

The mean age of the subjects was 21.7 years (SD = 2.7); 38.5% were male and 61.5% were female. Students' perceptions of learning, students' perceptions of teachers, students' academic self-perceptions, students' perceptions of atmosphere, students' social self-perceptions and total DREEM score were 21.2/48, 24.2/44, 15.8/32, 23.8/48, 14.5/28 and 99.6/200, respectively. There was no significant difference between male and female students in educational environment subscales, but there were significant differences between students enrolled on a basic sciences and pathophysiology course and those enrolled on a clinical course in terms of perceptions of learning, academic self-perceptions, perceptions of atmosphere and overall perceptions of educational environment (p < 0.05). The latter group rated each of the aforementioned aspects more highly than the students studying basic science and pathophysiology.

**Conclusion:**

Overall, respondents assessed the educational environment as average. Therefore, improvements are required across all five domains of the educational environment.

## Background

Recently, there have been dramatic changes in medical education world-wide [1], causing students' perceptions of their educational environment to receive special attention [2]. The quality of the educational environment is indicative of the effectiveness of an educational program. Educational environment sub-scales correlate positively with academic success and satisfaction with educational programs [3-6].

Students' perceptions of the educational milieu can be a basis for implementing modifications and thus optimize the educational environment. Meaningful learning correlates positively with the students' perceptions of the educational environment, which impacts on students' learning experiences and outcomes. It influences how, why and what students learn [6,7]. It is possible to assess and modify the educational environment. Accordingly, it is essential to utilize appropriate methods and instruments to assess it [1].

One method for assessing the educational environment is to evaluate students' perceptions of that environment. The Dundee Ready Education Environment Measure (DREEM) is routinely used to assess the educational climate, particularly in the realm of health and medicine [1]. This tool can be used to highlight the strengths and weaknesses of an educational institution, compare the performance and effectiveness of different medical schools, and make comparisons among students in different years of study and differences between the genders [6,7]. In addition, this instrument is used to help modify the curriculum, comparing past and present curricula and evaluating the efficacy of a university program [8,9]. It can help medical and health schools to recognize their educational priorities and introduce more effective measures as a result. Furthermore, it enables institutions to compare their performances and productivities with their peers, which can be educationally insightful [10]. Till utilized DREEM to make a comparison between the actual educational climate as perceived and experienced by students and the ideal one; the findings can be used to implement improvements in educational settings [11]. Students' perceptions of the educational climate may be swayed by the growing diversity of the student population, educational facilities and equipment, their expectations and other circumstances of the university, and this highlights the importance of assessing students' perceptions of their educational climate with a view to optimizing education.

In Iran, a general medicine course lasts seven years, almost half of which comprises basic sciences and pathophysiology course and the remainder clinical sciences course. Like many other medical schools in Iran, Hormozgan University of Medical Sciences employs a traditional educational system and curriculum, based chiefly on a teacher-centered and hospital-based approach. The curriculum, which comprises lectures in addition to specialized and practical courses, does not involve problem solving and is not particularly student-centered. Learning, perceived by students as a one-way transmission of information, is predominantly evaluated by summative final exams at the end of the course. The students' perceptions of this educational climate can shed light on the weaknesses and strengths of the system. To the best of our knowledge, no previous research has used the DREEM to assess perceptions of students regarding educational environment at medical schools in Iran. The aim of this study was to assess the perceptions of medical students concerning their educational environment at Hormozgan University of Medical Sciences in Iran.

## Methods

### Participants

This cross-sectional study was conducted at Hormozgan University of Medical Sciences in Bandar Abbas, south Iran, in 2009. No ethical issues were encountered during the course of this study. The study was approved by the Medical Ethics Committee of Hormozgan University of Medical Sciences.

The target population included students studying general medicine at the university. The school has approximately 350 general medicine students, but not all were present studying at the University at the time of the study. Thus, the questionnaires were distributed to 210 medical students; the maximum number of students who were present at the time of study. Twenty eight students were excluded as they did not return the questionnaire or did not complete it. Therefore, 182 questionnaires were analyzed (response rate = 86.6%).

### Measures

Medical students' perceptions of the educational environment were assessed by DREEM, a widely-used tool for gathering information about the educational environment in medical institutions. It was originally developed at Dundee University and has been validated as a universal diagnostic inventory for assessing the quality of the educational environment at different institutions. DREEM contains 50 statements concerning a range of topics directly relevant to the educational climate. The respondents were asked to read each statement and to respond using a five-point Likert scale ranging from strongly agree to strongly disagree. Items were scored as follows: 4 for strongly agree, 3 for agree, 2 for uncertain, 1 for disagree and 0 for strongly disagree. However, negative statements were scored in reverse. On this scale, a higher score indicates a more positive evaluation.

The 50-item DREEM has a maximum score of 200, indicating the ideal educational environment. It consists of the following five subscales:

• Students' Perceptions of Learning (12 questions, maximum score: 48)

• Students' Perceptions of Teachers (11 questions, maximum score: 44)

• Students' Academic Self-Perceptions (8 questions, maximum score: 32)

• Students' Perceptions of Atmosphere (12 questions, maximum score: 48)

• Students' Social Self-Perceptions (7 questions, maximum score: 28)

The DREEM can be used to pinpoint specific strengths and weaknesses within the educational climate by analyzing the responses to individual items. Items that have a mean score of 3.5 or above are classed as 'real positive points'. Items with a mean of two or less should be examined more closely as they are indicative of problem areas. Items with a mean between two and three are aspects of the climate that could be enhanced.

Prior to data collection, DREEM was translated into Persian using a backward-forward translation technique. A panel of medical education experts translated DREEM items from English into Farsi and then it was back-translated into English. Minor translation adjustments were carried out until the two versions (Farsi/English formats) were comparable. The reliability coefficient for each subscale was calculated using Cronbach's alpha. Cronbach's alpha for the totality of items was 0.91, which indicates high internal consistency. Cronbach's alpha values for students' perceptions of learning, students' perceptions of teachers, students' academic self-perceptions, students' perceptions of atmosphere and students' social self-perceptions were 0.80, 0.67, 0.70, 0.74 and 0.64, respectively.

Following clear instructions and clarifying the aim of the study, the questionnaires were distributed to the students. The basic sciences and pathophysiology course students did not complete three of the DREEM questions related to clinical contact. These items include: the teachers are patient with patients, the teachers have good communication skills with patients, and the atmosphere is relaxed during the ward teaching.

### Data analysis

Data were analyzed using SPSS16 software. Analysis of the data included comparisons of the mean scores of DREEM subscales, comparing male and female students, and comparing different courses. The Mann-Whitney test was used to determine statistically significant differences. In this study p < 0.05 was considered statistically significant.

## Results

The mean age of the participants in the study was 21.7 years (SD = 2.7), ranging from 18 to 30 years. Male and female students accounted for 38.5% and 61.5% of the responding sample, respectively. Students enrolled on basic sciences and pathophysiology course accounted for 57.7% of the respondents and the remaining 42.3% were enrolled on clinical course.

The overall score for students' perceptions of the educational environment was 99.6 (out of 200). Table 1 shows the mean scores for five subscales.

**Table 1:** Mean (SD) DREEM domain scores for participants

Domain	Mean	SD
Students' Perceptions of Learning (Max = 48)	21.2	7.1
Students' Perceptions of Teachers (Max = 44)	24.2	4.8
Students' Academic Self-Perceptions (Max = 32)	15.8	4.9
Students' Perceptions of Atmosphere (Max = 48)	23.8	6.8
Students' Social Self-Perceptions (Max = 28)	14.5	4.2
Total DREEM score (Max = 200)	99.6	22.9

Regarding the students' perceptions of learning, items 16 (the teaching helps to develop my competence) and 24 (the teaching time is put to good use) received mean scores greater than two (Table 2).

**Table 2:** Mean (SD) DREEM item scores for participants (Max = 4)

Items	Mean	SD
**Students' Perceptions of learning**		
1. I am encouraged to participate in class	1.9	1.1
7. The teaching is often stimulating	1.8	0.99
13. The teaching is student centered	1.7	1.01
16. The teaching helps to develop my competence	2.1	1.01
20. The teaching is well focused	2.0	0.99
22. The teaching helps to develop my confidence	1.7	0.97
24. The teaching time is put to good use	2.1	1.06
25. The teaching over-emphasizes factual learning	1.6	0.89
38. I am clear about the learning objectives of the course	1.9	1.1
44. The teaching encourages me to be an active learner	1.7	1.1
47. Long term learning is emphasized over short term learning	1.4	1.1
48. The teaching is too teacher-centered	1.5	1.04
**Students' perceptions of teachers**		
2. The teachers are knowledgeable	2.4	0.89
6. The teachers are patient with patients	2.1	0.76
8. The teachers ridicule the students	2.3	1.08
9. The teachers are authoritarian	2.1	1.01
18. The teachers have good communication skills with patients	2.2	0.62
29. The teachers are good at providing feedback to students	2.1	0.96
32. The teachers provide constructive criticism here	1.8	1.01
37. The teachers give clear examples	2.4	1.01
39. The teachers get angry in class	2.3	0.98
40. The teachers are well prepared for their classes	2.3	1.01
50. The students irritate the teachers	2.3	1.07
**Students' academic self-perceptions**		
5. Learning strategies which worked for me before continue to work for me now	1.6	1.02
10. I am confident about my passing this year	2.8	1.04
21. I feel I am being well prepared for my profession	1.9	1.1
26. Last year's work has been a good preparation for this year's work	1.8	1.03
27. I am able to memorize all I need	1.8	1.1
31. I have learned a lot about empathy in my profession	1.7	1.1
41. My problem solving skills are being well developed here	2.1	1.02
45. Much of what I have to learn seems relevant to a career in healthcare	2.1	1.2
**Students' perceptions of atmosphere**		
11. The atmosphere is relaxed during the ward teaching	1.9	0.82
12. This school is well timetabled	1.2	1.03
17. Cheating is a problem in this school	2.2	1.2
23. The atmosphere is relaxed during lectures	2.4	1.03
30. There are opportunities for me to develop interpersonal skills	2.0	1.1
33. I feel comfortable in class socially	2.2	1.1
34. The atmosphere is relaxed during seminars/tutorials	2.0	1.03
35. I find the experience disappointing	2.2	1.2
36. I am able to concentrate well	1.9	1.2
42. The enjoyment outweighs the stress of the course	2.5	1.2
43. The atmosphere motivates me as a learner	1.5	1.1
49. I feel able to ask the questions I want	1.8	1.1
**Students' social self-perceptions**		
3. There is a good support system for students who get stressed	1.1	1.02
4. I am too tired to enjoy the course	1.6	1.2
14. I am rarely bored on this course	2.0	1.1
15. I have good friends in this school	2.8	1.08
19. My social life is good	2.7	1.03
28. I seldom feel lonely	2.1	1.1
46. My accommodation is pleasant	2.2	1.3

In terms of students' perceptions of teachers, all items except number 32 (the teachers provide constructive criticism here) received mean scores greater than two (Table 2).

Regarding the students' academic self-perceptions, items 10 (I am confident about my passing this year), 41 (my problem solving skills are being well developed here) and 45 (much of what I have to learn seems relevant to a career in healthcare) received mean scores greater than two.

In terms of students' perceptions of atmosphere, five items received mean scores greater than two (Table 2).

Concerning students' social self-perceptions, items 15 (I have good friends in this school), 19 (my social life is good), 28 (I seldom feel lonely) and 46 (my accommodation is pleasant) received mean scores greater than two (Table 2).

There was no significant difference between the genders in any of the educational environment subscales (Table 3), but there were significant differences between students enrolled on the basic sciences and pathophysiology course and those studying the clinical course in terms of perceptions of learning, academic self-perceptions, perceptions of atmosphere and overall perceptions of the educational environment (p < 0.05); the latter group rated the aforementioned aspects more highly than those enrolled on the basic sciences and pathophysiology course (Table 4).

**Table 3:** Comparison of DREEM domain scores for male and female students (Mann-Whitney)

Subscales	Male Mean (SD)	Female Mean (SD)	p
Students' Perceptions of Learning (Max = 48)	22.1 (6.6)	20.6 (7.1)	0.08
Students' Perceptions of Teachers (Max = 44)	23.8 (4.6)	24.4(5.1)	0.37
Students' Academic Self-Perceptions (Max = 32)	16.6 (4.2)	15.3 (5.3)	0.06
Students' Perceptions of Atmosphere (Max = 48)	24.7 (6.1)	23.2 (7.3)	0.13
Students' Social Self-Perceptions (Max = 28)	14.9 (3.6)	14.2 (4.5)	0.39
Total DREEM score (Max = 200)	102.3 (20.2)	97.9 (24.3)	0.13

**Table 4:** Comparison of DREEM domain scores for students studying basic sciences and those enrolled on a clinical course (Mann-Whitney)

Subscales	Basic science Mean (SD)	Clinical Mean (SD)	p
Students' Perceptions of Learning (Max = 48)	20.3 (7.6)	22.3 (5.8)	0.04*
Students' Perceptions of Teachers (Max = 44)	23.8 (5.3)	24.6 (4.2)	0.34
Students' Academic Self-Perceptions (Max = 32)	14.7 (4.9)	17.4 (4.5)	0.001*
Students' Perceptions of Atmosphere (Max = 48)	22.3 (6.8)	25.9 (6.3)	0.000*
Students' Social Self-Perceptions (Max = 28)	14.4 (4.2)	14.6 (4.2)	0.63
Total DREEM score (Max = 200)	95.7 (24.1)	104.9 (20.1)	0.008*

* significant

## Discussion

There has been growing interest and concern about the role of the learning environment in medical education. Educational environment is one of the most important factors in determining the success of an effective curriculum [12].

The results presented herein revealed a mean overall score of 99.6/200 for the DREEM items. According to the practical guide of McAleer and Roff [13], a mean score between 50 and 100 indicates potential problems. In medical schools with a traditional system, scores are found to be below 120; however, in modern, student-centered ones, the mean score is generally much higher [14,15]. In a survey carried out at a medical school in England that used DREEM [14], the mean score was calculated as 124/200. In another investigation concerning eight teaching hospitals in Birmingham, England [15], the mean score was 139/200. These values were higher than in the present study. One explanation is that these Universities have modern systems, while the medical school of Hormozgan University has a traditional system. In a similar study carried out in Trinidad [8], a mean score of 109.9/200 was obtained. In King Abdul Aziz University, Saudi Arabia [10], a mean score of 102/200 was reported and this is close to the mean presented in the current study. The similarity of the results could be due to similarity in the educational systems.

In the present study, the scores were: students' perceptions of learning 21.2/48, perceptions of teachers 24.2/44, academic self-perceptions 15.8/32, perceptions of atmosphere 23.8/48 and social-self perceptions 14.5/28. In King Abdul Aziz University, Saudi Arabia [10], the scores obtained for the aforementioned subscales were 22/48, 24/44, 17/32, 23/48 and 15/28, respectively. Although there are some non-significant and subtle differences between the two schools, it can be concluded that the findings were similar, and might be explained by the traditional system prevailing in these universities. In an Indian medical school with a traditional system [12], the DREEM subscales scores were higher than those of the present study. In a study conducted by Al-Hazimi et al. on three traditional and one innovative medical schools, the mean scores for the traditional medical schools were lower than the innovative one. Students from traditional schools rated their learning and teaching environment significantly lower than their counterparts in the innovative medical school. Similarly, they rated their academic self-perceptions, social-self perceptions and their atmosphere lower than students from the innovative medical school [9]. According to the results of this study and the practical guide of McAleer and Roff [13], regarding the students' perceptions of learning, teaching is viewed negatively; regarding their perceptions of teachers, the school is moving in the right direction; regarding their academic self-perceptions, there are many negative aspects; regarding their perceptions of atmosphere, there are many issues that require change; regarding the students' social-self perceptions, the school is not too bad.

Furthermore, no item received a mean score ≥3.5. A mean score ≥3.5 indicates particularly positively-rated items. Twenty four items received mean scores between two and three. These items are aspects of the educational environment that could be enhanced [13]. Twenty six items received mean scores ≤ 2, indicating problem areas [13]. The three most highly scored items were: item 10 (I am confident about my passing this year), which received a score of 2.8; item 15 (I have good friends in this school), with a score of 2.8; and item 19 (my social life is good), with a score of 2.7. The four items that received the lowest scores were: item 3 (there is a good support system for students who get stressed); item 12 (this school is well timetabled); item 47 (long-term learning is emphasized over short-term learning); and item 48 (the teaching is too teacher-centered), which received scores of 1.1, 1.2, 1.4 and 1.5, respectively. The low scores are a cause for concern. In a medical school in England [14], low scores were found to be related to the following items: there is a good support system for students who get stressed, this school is well timetabled, the teachers are good at providing feedback to students, and I am able to memorize all I need. The low scores given to the above items are similar to the findings in the present study. In King Abdul Aziz University, Saudi Arabia [10], the item "There is a good support system for students who get stressed" had a poor score of 0.9, which is echoed in our study. A low rating of this item would refer to a perceived lack of support available to those students who get stressed.

Students on the clinical course rated the educational environment more highly than students in the basic sciences and pathophysiology course. One possible explanation is that the basic sciences and pathophysiology course students did not complete three items of DREEM questions related to clinical contact. In an Indian medical school [12], the total DREEM domain score was higher for first year students than students receiving clinical teaching, whereas in this study, students on the clinical course received a higher total DREEM score than students studying basic sciences and pathophysiology during the first year.

In addition, none of the subscales in our study indicated a significant difference with respect to students' gender. In contrast, in the studies by Fidelma [14] and Bassaw [8], females rated the educational milieu higher than their male counterparts.

## Conclusion

In conclusion, participants assessed the educational environment as average. Regarding the students' perceptions of learning, teaching was viewed negatively; regarding their perceptions of teachers, the school is moving in the right direction; regarding their academic self-perceptions, there are many negative aspects; regarding their perceptions of the atmosphere, there are many issues that require change; and regarding the students' social-self perceptions, the school is not too bad. Therefore, improvements are required across all five domains of the educational environment.

## Abbreviations

DREEM: The Dundee Ready Education Environment Measure

## Competing interests

The authors declare that they have no competing interests.

## Authors' contributions

TA designed and conducted the study, performed statistical analysis and drafted the manuscript. IF participated in data collection, assisted with drafting the manuscript and edited it. All authors read and approved the final manuscript.

## Pre-publication history

The pre-publication history for this paper can be accessed here:

http://www.biomedcentral.com/1472-6920/10/87/prepub
